# An online behavioral self-help intervention rapidly improves acute insomnia severity and subjective mood during the coronavirus disease-2019 pandemic: a stratified randomized controlled trial

**DOI:** 10.1093/sleep/zsae059

**Published:** 2024-03-02

**Authors:** Greg J Elder, Nayantara Santhi, Amelia R Robson, Pamela Alfonso-Miller, Kai Spiegelhalder, Jason G Ellis

**Affiliations:** Northumbria Sleep Research, Northumbria University, Newcastle upon Tyne, UK; Northumbria Sleep Research, Northumbria University, Newcastle upon Tyne, UK; Northumbria Sleep Research, Northumbria University, Newcastle upon Tyne, UK; Northumbria Sleep Research, Northumbria University, Newcastle upon Tyne, UK; Department of Psychiatry and Psychotherapy, Faculty of Medicine, Medical Center - University of Freiburg, Freiburg, Germany; Northumbria Sleep Research, Northumbria University, Newcastle upon Tyne, UK

**Keywords:** COVID-19, clinical trials research, insomnia, online intervention, stress

## Abstract

**Study Objectives:**

Stressful life events, such as the coronavirus disease-2019 (COVID-19) pandemic, can cause acute insomnia. Cognitive behavioral therapy for acute insomnia is effective but is both time and resource-intensive. This study investigated if an online behavioral self-help intervention, which has been successfully used alongside sleep restriction for acute insomnia, reduced insomnia severity and improved mood in acute insomnia. This study also assessed good sleepers to explore if a “sleep vaccination” approach was feasible.

**Methods:**

In this online stratified randomized controlled trial, 344 participants (103 good sleepers and 241 participants with DSM-5 acute insomnia) were randomized to receive the intervention/no intervention (good sleepers) or intervention/intervention after 28 days (poor sleepers). Insomnia severity was assessed using the ISI (primary outcome), and anxiety and depression using the GAD-7/PHQ-9 (secondary outcomes) at baseline, 1 week, 1 month, and 3-month follow-up.

**Results:**

In people with acute insomnia, relative to baseline, there were significant reductions in ISI (*d*_*z*_ = 1.17), GAD-7 (*d*_*z*_ = 0.70), and PHQ-9 (*d*_*z*_ = 0.60) scores at 1-week follow-up. ISI, GAD-7, and PHQ-9 scores were significantly lower at all follow-up time points, relative to baseline. Subjective diary-derived sleep continuity was unaffected. No beneficial effects on sleep or mood were observed in good sleepers.

**Conclusions:**

An online behavioral self-help intervention rapidly reduces acute insomnia severity (within 1 week), and benefits mood in people with acute insomnia. These beneficial effects are maintained up to 3 months later. Although the use of the intervention is feasible in good sleepers, their subjective sleep was unaffected.

**Clinical Trial registration:**

Testing an early online intervention for the treatment of disturbed sleep during the COVID-19 pandemic; prospectively registered at ISRCTN on 8 April 2020 (identifier: ISRCTN43900695).

## Graphical Abstract

**Graphical Abstract:**
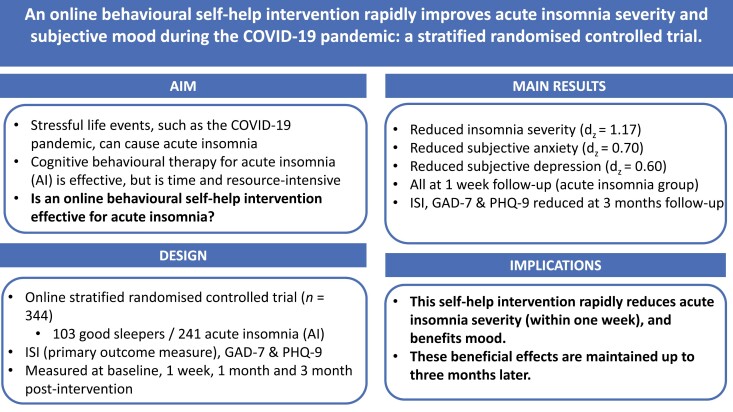

**Statement of Significance**

Stressful life events, such as the coronavirus disease-2019 pandemic, can cause acute insomnia. Cognitive behavioral therapy for acute insomnia is effective but is both time and resource-intensive. The results of this study indicate that a simple and easy-to-use online self-help behavioral leaflet can very rapidly improve insomnia symptoms, and subjective anxiety and depression, in people with acute insomnia. Importantly, these improvements are maintained at up to 3 months follow-up. This intervention therefore represents an effective and low-cost, non-pharmacological treatment method for acute insomnia. This intervention could potentially be rolled out at scale in order to prevent the transition from acute insomnia to insomnia disorder.

## Introduction

Insomnia disorder is a highly prevalent sleep disorder within industrialized societies, with approximately 6%–10% of the population meeting criteria for insomnia disorder, and a much higher number (up to 48% of the population) reporting insomnia symptoms [1, 2]. In addition to being highly prevalent, insomnia disorder is associated with multiple negative physical and psychological health outcomes, and a significant level of economic costs [3–6]. Theoretical models of insomnia (e.g. the “3P” model [7, 8]) indicate that stressful life events can result in acute insomnia, which is clinically significant short-term insomnia [7–9]. Acute insomnia is highly prevalent with annual incidence rates as high as 40% being reported [10, 11]. Acute insomnia can lead to the development of insomnia disorder, as theoretically, developing a preoccupation with sleep can lead to long-term sleep disturbances through behavioral conditioning [9]. Indeed, it has been shown that approximately 7% of people with acute insomnia develop insomnia disorder, and a further 20% of individuals who exhibit subjective sleep disturbances, which are not of a sufficient severity to be considered acute insomnia, go on to develop insomnia disorder, albeit at a slower rate [11].

Stress refers to experiences that are considered to be emotionally or physiologically demanding, and the adaptive physiological response [12, 13] can negatively impact sleep [13, 14]. A variety of naturalistic studies have demonstrated that stressful events, in the form of earthquakes, hurricanes, and war, can disrupt sleep [15–17]. The coronavirus disease-2019 (COVID-19) pandemic represents a stressful event, being a stressor of unknown duration accompanied by a range of societal and lifestyle changes [18]. Recent work has indicated that the pandemic is considered to be a stressful and traumatic event [19, 20]; for instance, population survey studies have demonstrated that subjective levels of stress have increased during the course of the pandemic [21, 22], with an estimated 20%–30% of the general population reporting experiencing elevated levels of stress [23, 24]. Cross-sectional work has demonstrated that the pandemic has had a negative impact on sleep [25–27], and that pandemic-related sleep problems are also associated with anxiety and depression [28]. A recent meta-analysis has estimated that the global prevalence of sleep disturbances related to COVID-19 is approximately 40% [29]. Taken together, the COVID-19 pandemic is extremely likely to cause sleep disturbances and trigger acute insomnia [30]. Given the high health and economic costs that is associated with insomnia disorder [3–6], early interventions are necessary to prevent the transition from acute insomnia to insomnia disorder. This is important as symptoms of insomnia have been shown to continue even after the emotional consequences of the initial trigger have abated [31]. Additionally, the early treatment of acute insomnia may also prevent the development of later psychiatric comorbidities, such as depression, which occur alongside insomnia disorder [32, 33].

Pharmacological treatments, including benzodiazepines, are effective short-term treatments for insomnia disorder [34]. However, these agents are associated with side effects including dependency and next-day drowsiness, and with negative health outcomes including increased mortality [35–37]. Consequently, some pharmacological treatments may be particularly unsuitable for certain groups, including older adults [38, 39]. For this reason, non-pharmacological treatments are likely to be advantageous as an early intervention at the acute insomnia stage. Non-pharmacological treatments are effective for insomnia disorder, and one such treatment, cognitive behavioral therapy for insomnia (CBT-I), which is a structured psychotherapy with the aim of modifying cognitions and behaviors, which contribute to the maintenance of insomnia [40], is highly effective [41]. Although CBT-I is a recommended first-line treatment for insomnia disorder [41], the widespread delivery of this intervention is predominantly limited by the lack of qualified CBT-I providers, and also by the high levels of attrition that are observed during treatment [42]. Although CBT-I can be delivered digitally and can be done using automated methods, addressing the issue of provision, digital CBT-I typically involves multiple structured treatment sessions, which are delivered over a period of several weeks [43, 44] and require the appropriate time commitment from patients. Moreover, adherence to digital CBT-I remains an issue [41]. For this reason, shorter interventions, which rely upon the principles of CBT-I, may be able to overcome these limitations whilst maintaining their effectiveness.

It has previously been shown that a self-help leaflet, which outlines the components of stimulus control, cognitive control, and imagery distraction techniques, significantly reduced insomnia severity when delivered alongside a single (“one shot”) 60–70 minute CBT-I session focused on sleep restriction in a group of individuals with acute insomnia [42]. Follow-up studies have demonstrated the effectiveness of this intervention when delivered in a group format, and to an adult male prison population [40, 45]. While previously the leaflet was delivered alongside sleep restriction, recently it has been demonstrated that sleep extension may not be a feature of acute insomnia; therefore, this component may not be required therapeutically in this context [46, 47]. Additionally, as maladaptive sleep-related behaviors and cognitions are suggested to drive the transition from acute insomnia to insomnia disorder [9], this suggests that the self-help leaflet alone, which largely addresses sleep-related dysfunctional cognitions, may be an effective short intervention for acute insomnia in the context of the COVID-19 pandemic.

A specific advantage of this interventional approach is that it is well-suited to a large-scale online delivery model, by reaching a large number of individuals in the context of a major stressful event. In further support of the online delivery model, it has been shown that internet-based CBT-I is effective, with similar effect sizes when compared to face-to-face delivery, with the specific advantage of lower costs [48, 49]. Therefore, this intervention may be an extremely promising method of reducing acute insomnia severity; however, it is untested.

It is also not clear how long the effectiveness of the self-help leaflet alone would be maintained for. This intervention may also benefit good sleepers by promoting the avoidance of sleep-related behaviors and cognition, therefore potentially reducing the impact of, or preventing, stress-related sleep disturbances [30]; theoretically, it is possible to protect good sleep in this context [9]. However, this intervention has not been used for this purpose and the feasibility, and effectiveness, of this concept is also as yet untested.

The primary aim of the present study was to assess the short-term and longer-term effectiveness of an online behavioral intervention for individuals with acute insomnia, in the context of an ongoing, stressful, major life event. Secondary aims of the present study included assessing if the intervention could reduce subjective anxiety and depression in individuals with acute insomnia. An additional secondary aim was to test the feasibility, and effectiveness, of the intervention on sleep and mood in good sleepers. It was hypothesized that the intervention would reduce insomnia severity in participants with acute insomnia: (1) in the short-term (1-week post-intervention) and (2) in the longer-term (1- and 3 months post-intervention).

## Materials and Methods

### Trial design

The study was a stratified randomized controlled trial, involving self-reported good sleepers and participants with acute insomnia. The study was delivered online (Qualtrics, Provo, UT) between August 2020 and April 2022, and was ended as the required sample size was obtained. After providing informed consent, acute insomnia participants were randomized into an intervention or waitlist group (1:1 ratio), and good sleepers were randomized into an intervention or no intervention group (1:1 ratio).

The primary aim of this study was to specifically assess the short-term, and longer-term, effectiveness of the intervention in participants with acute insomnia, and the participant dropout rate of this specific intervention is not known when delivered online [50]. Therefore, the primary outcomes were assessed by comparing post-intervention ISI scores relative to baseline scores in the acute insomnia group only. The purpose of the waitlist poor sleeper group was to assess levels of participant dropout to inform the use of waitlist control studies using this intervention in future [50]. Allocation sequences were automatically generated by Qualtrics software without any influence from any member of the research team. The full study protocol has been published previously [50].

**Figure 1.**
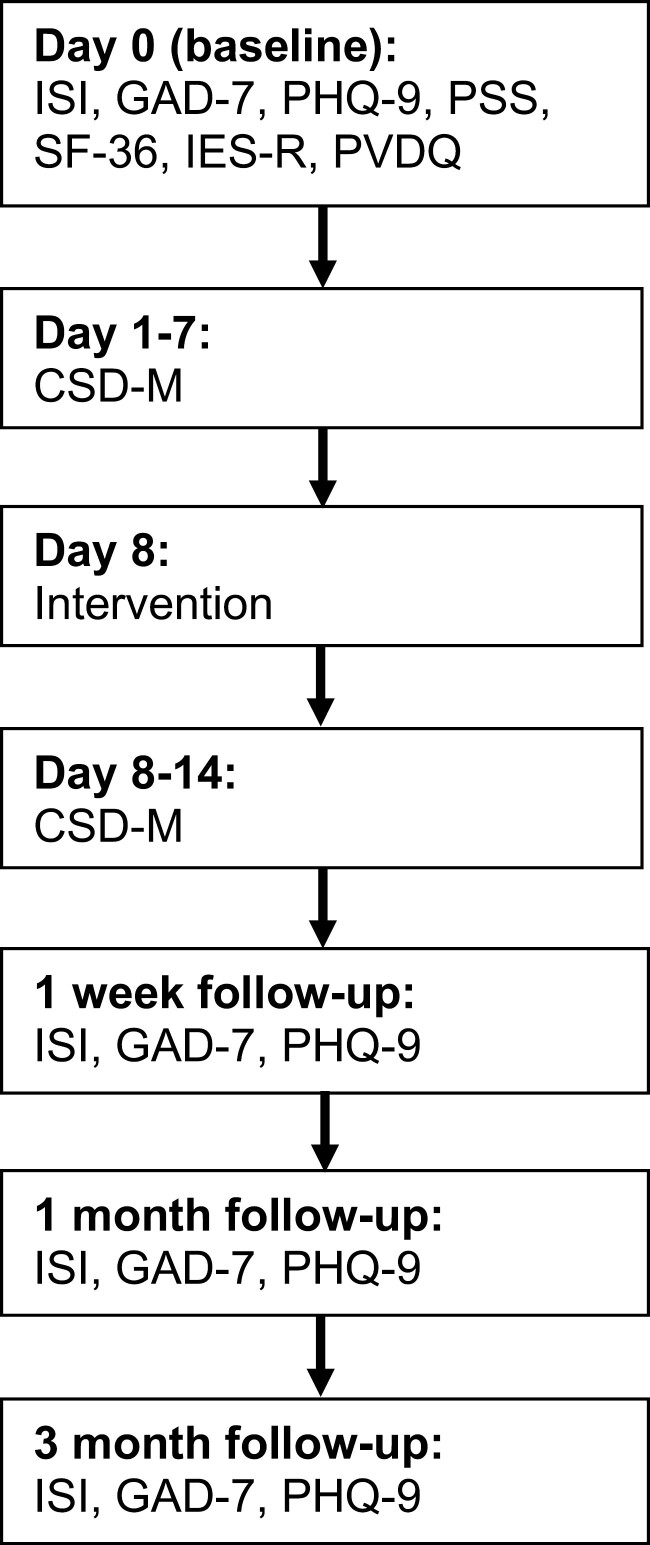
Study procedure.

All participants completed baseline assessments (day 0). After a pre-intervention sleep monitoring period (days 1–7), participants were given the intervention on day 8 (acute insomnia and good sleeper intervention groups only). After a post-intervention sleep monitoring period (days 8–14), participants were followed up at 1 week, 1 month, and 3 months post-intervention. Waitlist acute insomnia participants repeated assessments (measures of insomnia severity, self-reported anxiety and depression, and perceived stress) at day 28 and were given the intervention on day 36. Good sleeper participants who did not receive the intervention completed follow-up stages at the equivalent time point (1 week, 1 month, and 3 months). Details of the assessments which were completed by both groups are provided in Figure 1. The study was prospectively registered (ISRCTN43900695; registered April 8, 2020). All participants provided electronic informed consent and the study was approved by the Northumbria University Department of Psychology Ethics Committee.

### Participants

Both self-reported people with acute insomnia and good sleepers were eligible to take part in the study, and participants were recruited using social media advertisements and university news announcements. Participants were eligible to take part if they were aged ≥18 years and if they had a sufficient level of English comprehension to understand and complete all study measures. Acute insomnia participants were required to meet criteria for acute insomnia, as defined by the Diagnostic and Statistical Manual of Mental Disorders (DSM-5) [51], where they were required to report: (1) difficulties falling asleep, staying asleep, or awakening too early for at least three nights per week, for a time period of between 2 weeks and 3 months, and (2) the occurrence of distress or impairment. Both criteria needed to occur despite the individual having had an adequate opportunity for sleep. Healthy good sleepers were self-defined on the basis of not reporting any current sleep problems. As the aim of the study was to make the intervention available to as many participants as possible, potentially eligible participants were not prescreened before providing informed consent.

Individuals were informed that they were not eligible to participate in the present study if they self-reported: (1) a chronic sleep problem which had persisted for >3 months before the date of participation, (2) if they were actively using another treatment specifically to treat a sleep disturbance or disorder, (3) a reported history of head injury, diagnosis of schizophrenia, epilepsy or personality disorder (this was included because the feasibility of this intervention has not been tested in those specific groups of individuals, and this may have influenced the subsequent effectiveness of the “Distract” component, as a specific pre-sleep cognitive activity is included). Participants were not included or excluded on the basis of any baseline assessment score, or based on the presence of any comorbidities.

### Measures

Insomnia severity was assessed using the Insomnia Severity Index (ISI [52]), which is a seven-item measure that quantifies the nature, severity, and effect of insomnia. The ISI provides scores ranging from 0 to 28, where high scores represent more severe insomnia. As per previous work [42], the ISI was modified in order to assess insomnia severity during the previous week, as opposed to the preceding month. The ISI is a reliable and valid measure for quantifying perceived insomnia severity [49]. Subjective sleep continuity was measured using the Consensus Sleep Diary (CSD-M [53]). Standard sleep measures, including total sleep time (TST), time in bed (TIB), number of awakenings (NWAK), wake after sleep onset (WASO), sleep onset latency (SOL), and sleep efficiency (SE%) were derived from completed diaries.

The Patient Health Questionnaire (PHQ-9 [54]) and the Generalized Anxiety Disorder Questionnaire (GAD-7 [55]) were used to assess self-reported symptoms of depression and anxiety, respectively, and were modified to assess the previous week at follow-up stages. As a measure of subjective stress, participants completed the Perceived Stress Scale (PSS [56]) which assesses the extent to which 14 life situations are perceived as being stressful in the preceding month. General health status and health-related quality of life were measured using the 36-Item Short Form Survey Instrument (SF-36 [57]). The SF-36 assesses eight domains, including physical function, role limitations because of physical health problems, bodily pain, general health perceptions, general mental health, role limitations because of mental health problems, social functioning, and vitality.

Participants were also asked to indicate their levels of subjective distress, and perceived infection susceptibility, specifically in relation to COVID-19. Participants completed the Impact of Event Scale-Revised (IES-R [58]), which is a measure that quantifies the subjective level of distress caused by a specific stressful or traumatic life event. The IES-R asks participants to indicate how distressed they are with regard to specific difficulties during the previous week (e.g. feeling irritable or angry; being jumpy or easily startled, feeling watchful and on guard); higher IES-R scores represent greater levels of distress. Participants also completed the Perceived Vulnerability to Disease questionnaire (PVD [59]), which measures the perceived susceptibility to infectious diseases, and emotional discomfort in situations with the potential for high infection transmission (e.g. being bothered when people sneeze without covering their mouths; being anxious around sick people; believing that they will catch an illness that is “going around”). The PVD has two separate subscales (Germ Aversion and Perceived Infectability); higher PVD subscale scores represent greater levels of perceived infection susceptibility and emotional discomfort, respectively. Participants were asked to complete the IES-R and PVD specifically in relation to the ongoing COVID-19 pandemic. Finally, participants were asked to self-report whether they had been diagnosed with or if they had demonstrated symptoms of COVID-19.

### Intervention

Eligible participants were provided with an online two-page sleep self-help leaflet in an electronic file format (PDF). The self-help leaflet has been previously used [42], and explains the principles of Stimulus Control, Cognitive Control and Imagery Distraction techniques [60, 61], in the format of “three D’s” (“Detect,” “Detach,” and “Distract”) [42] in relation to stress-related sleep loss. Specifically, the “Detect” component provided participants with detailed instructions for the completion of subjective sleep diaries, with an explanation that diaries are useful tools for monitoring habitual sleep patterns. The stimulus control “Detach” component included basic sleep hygiene advice and pre-sleep relaxation guidance, and finally, the cognitive control and imagery distraction (“Distract”) component provided a specific pre-sleep task, which was designed to replace stressful thoughts with other pre-sleep cognitive activity, but does not encourage individuals to think about emotionally salient information [42]. The intervention was fully self-administered and there were no restrictions on the use of the leaflet: participants were permitted, and were specifically encouraged, to download, print, or take mobile phone screenshots of the leaflet. To access the self-help leaflet, it was necessary for participants to click a link, and this was electronically recorded.

### Sample size calculation

An a priori power analysis, which was conducted using G*Power 3.1 [62], indicated that a minimum of 54 acute insomnia participants were required to compare the primary outcome measure between baseline and 1-week follow-up, using a two-tailed repeated-measures *t*-test with an expected medium effect size of *d*_z_ = 0.50 (at 95% power; *α* = 0.05). This effect size is based on previous studies from our group which has used this intervention, where a medium-to-large (*d* = 0.64) effect was observed in people with acute insomnia and a large (*d*_z_ = 2.35) effect was observed in prison inmates with acute insomnia; in both cases, the ISI was the primary outcome measure [40, 42]. For good sleepers, an a priori power analysis indicated that 54 participants were required, on the basis of an expected interaction effect on the ISI, using a 2 (intervention vs. no intervention) × 2 (time point: baseline vs. 1-week follow-up) mixed analysis of variance (ANOVA) with an expected medium effect size of *f*^2^_=_ 0.25 (at 95% power, expected correlation between repeated measures = 0.5; *α* = 0.05). As the study aimed to recruit an equal number of good sleeper and acute insomnia sleeper participants (total *n *= 108), allowing for an expected 15% overall participant attrition rate (*n *= 16), a total of 124 participants were required, with good sleeper and acute insomnia sleepers recruited at a 1:1 ratio (*n *= 62 per group).

Over-recruitment was permitted to protect against the potential for a higher-than-anticipated participant dropout rate. Ethically, this was permissible as none of the groups received a harmful treatment, and treatment-as-usual in the UK for acute insomnia, which is delivered by a general practitioner in the first instance, typically involves sleep hygiene advice followed by pharmacological treatment [41, 63].

### Statistical analyses

Baseline (day 0 or, in the case of waitlist acute insomnia participants, the equivalent time point of day 28) sleep data were compared between groups using independent-sample *t*-tests (in the case of continuous data) or chi-square tests (in the case of categorical data). Effect sizes (*d, d*_z_, or η^2^_p_) are reported where appropriate.

### Primary outcome measure

The primary outcome measure was the ISI. As the main aim of the study was to test the effectiveness of the intervention in poor sleepers, the short-term effectiveness was assessed by comparing baseline and 1-week post-intervention ISI scores, in poor sleepers, using a repeated-measures *t*-test. The longer-term effectiveness of the intervention was assessed by comparing ISI scores, in poor sleepers, between baseline and 1 week, 1 month, and 3-month follow-up time points using a repeated measures ANOVA. These analyses were pre-specified and statistical significance was assessed at the threshold of *p *= 0.05.

### Secondary outcome measures

In poor sleepers, subjective sleep continuity measures (TIB, TST, SE%, SOL, NWAK, and WASO) were compared pre-intervention and post-intervention (averaged days 1–7 compared to averaged days 8–14) using repeated-measures *t*-tests, adjusted for multiple comparisons (adjusted *p*-value* *= 0.008). SE% values of > 100% were removed prior to all subjective sleep diary analyses. GAD-7 and PHQ-9 scores were compared in the short-term (baseline vs. 1-week follow-up) using repeated-measures *t*-tests, and longer-term (baseline vs. 1 week, 1 month, and 3 months follow-up) using a one-way ANOVA.

In good sleepers, the short-term effectiveness was assessed by comparing participants who received the intervention relative to those who did not, by comparing ISI scores between baseline and 1-week follow-up using a 2 (group) × 2 (time point) mixed ANOVA. The longer-term effectiveness was assessed using a 2 (group) × 4 (time point: baseline, 1 week, 1 month, and 3-month follow-up) mixed ANOVA (*p*-values* *= 0.05). Subjective diary-derived sleep continuity, GAD-7, and PHQ-9 scores were also examined in the same manner as poor sleepers.

Additional exploratory analyses included the calculation of remission rates in acute insomnia participants (expressed as a percentage); remission was defined as cases where participants who had ISI scores of ≥ 10 at baseline, and ISI scores of < 10 at follow-up time points [64]. Transition rates were also calculated in good sleepers, and this was defined as cases where participants displayed ISI scores of ≥ 10 at follow-up time points (i.e. participants who transitioned from good sleep to poor sleep).

## Results

### Participant flow

**Figure 2.**
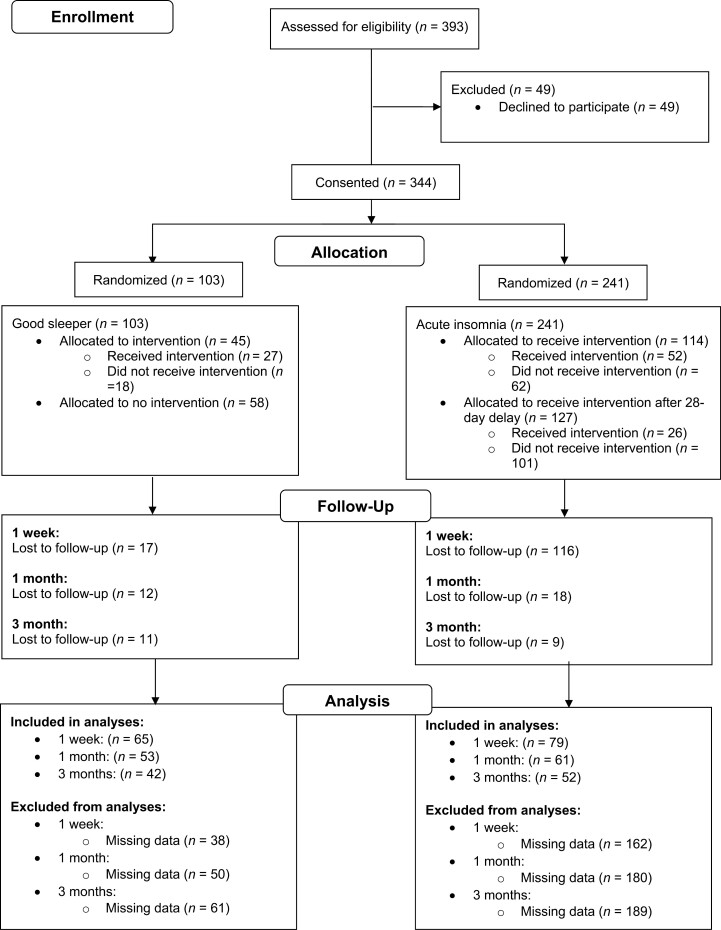
CONSORT participant flow diagram.

A total of 344 participants were entered into the study (103 good sleepers and 241 acute insomnia participants, M_age_ = 32.8 years, *SD*_age_ = 16.2 years). A CONSORT (Consolidated Standards of Reporting Trials [65]; Supplementary Material) flowchart is included in Figure 2.

Complete baseline ISI data were obtained for 277 participants (82 good sleepers and 195 acute insomnia). Complete ISI follow-up data were obtained at 1 week for 147 participants (68 good sleepers and 79 acute insomnia), at one month for 120 participants (58 good sleepers and 62 acute insomnia), and at 3 months for 99 participants (46 good sleepers and 53 acute insomnia).

Baseline demographic measures are reported in Table 1. Acute insomnia participants were significantly older than good sleepers, and also reported significantly higher levels of perceived stress (PSS), greater levels of distress in relation to the ongoing pandemic (IES-R), and perceived vulnerability to disease (PVDQ), as well as significant higher levels of insomnia severity (ISI), anxiety (GAD-7) and depression (PHQ-9; all *p*-values < .05; Table 1).

**Table 1.** Baseline Participant Data (*n* = 277)

	Good sleepers (*n* = 82)		Acute insomnia (*n* = 195)		
	Mean	*SD*	Mean	*SD*	*P*
Age (years)^a^	26.11	12.14	34.26	16.35	<.001
Gender (male/ female/ other; *n*/ %)	13(15.9)/ 68(82.9)/ 1(1.2)		23 (11.8)/ 171(87.7)/ 1(0.5)		.53
Positive COVID-19 symptoms (self-report; *n*/ %)	29 (35.4)		58 (29.7)		.36
Positive COVID-19 diagnosis (self-report; *n*/ %)	21 (25.6)		33 (16.9)		.10
PSS	19.18	6.32	22.75	6.79	<.001
SF-36 (general health %)	45.28	20.81	41.11	20.99	.13
IES-R^a^	24.67	16.44	39.31	19.48	<.001
PVDQ	55.80	14.68	61.81	13.70	.001
ISI	6.29	4.61	16.68	4.71	<.001
GAD-7	7.24	5.46	11.22	5.68	<.001
PHQ-9^a^	7.00	5.60	12.43	5.99	<.001

^a^(*n* = 276).

*Abbreviations*: GAD-7, 7-item Generalized Anxiety Disorder Questionnaire; IES-R, Impact of Event Scale-Revised; ISI, Insomnia Severity Index; PHQ-9, 9-item Patient Health Questionnaire; PSS, Perceived Stress Scale; PVDQ, Perceived Vulnerability to Disease Questionnaire.

### Participant dropout rates

The participant dropout rate for acute insomnia participants who received the intervention on Day 8 (*n *= 96 with completed baseline ISI), was 49.0%, 66.7%, and 77.1% at 1-week, 1-month, and 3-month follow-up periods, respectively. The acute insomnia participant dropout rate for those who were on the waitlist condition (*n *= 99 with completed baseline ISI), was 70.7%, 76.7%, and 80.8% at 1-week, 1-month, and 3-month follow-up time points. The participant dropout rates for good sleepers who did receive the intervention (*n *= 45) were 33.3%, 57.8%, and 60.0%, and for those good sleepers who did not receive the intervention (*n *= 37), dropout rates were 24.3%, 32.4%, 62.2% at 1 week, 1 month and 3-month follow-up time points. Participant dropout rates were significantly associated with participant group at 1 week (χ^2^(1, 277) = 33.93, *p < *.001; ϕ = 0.35), 1 month (χ^2^(1, 277) = 20.03, *p < *.001; ϕ = 0.27) and 3-month follow up (χ^2^(1, 277) = 11.74, *p < *.01; ϕ = 0.21) time points.

### Outcomes

#### Primary outcome measure.

In acute insomnia participants who received the intervention, comparisons of ISI scores between baseline and 1-week follow-up showed that in the short-term, insomnia severity significantly reduced, with a large effect size (*t*(77) = 8.72, *p < *.001; *d*_*z*_ = 1.17; Table 2). In the longer term, comparisons of ISI scores showed that there was a significant reduction in insomnia severity between baseline and all follow-up time points in those who received the intervention, as shown by the significant main effect of time point (*F*(2.19, 89.69) = 14.41, *p < *.001; η^2^_p_ = 0.26; Table 3). *Post hoc* comparisons indicated that relative to baseline, ISI scores at all follow-up time points (1 week, 1 month, and 3 months) had significantly reduced.

**Table 2.** Acute Insomnia Participant Insomnia Severity and Mood Comparisons: Baseline and 1-Week Follow-up (*n *= 78)

	Baseline		1 week follow-up			
	Mean	*SD*	Mean	*SD*	*P*	*d*_*z*_
ISI	16.32	5.10	12.03	4.73	<.001	1.17
GAD-7	10.44	6.33	7.40	4.70	<.001	0.70
PHQ-9^a^	11.39	5.88	8.89	5.18	<.001	0.60

^a^(*n* = 77).

*Abbreviations*: GAD-7, 7-item Generalized Anxiety Disorder Questionnaire; ISI, Insomnia Severity Index; PHQ-9, 9-item Patient Health Questionnaire.

**Table 3.** Acute Insomnia Participant Insomnia Severity and Mood Comparisons: Baseline and All Follow-up Time Points (*n *= 42)

	Baseline		1 week		1 month		3 month			
	Mean	*SD*	Mean	*SD*	Mean	*SD*	Mean	*SD*	*P*	η^2^_p_
ISI	15.00	4.83	11.52*	4.66*	10.43*	4.59*	10.33*	5.50*	<.001	0.26
GAD-7^a^	9.16	6.24	6.86*	4.82*	6.45*	5.28*	6.39*	5.28*	<.001	0.14
PHQ-9^b^	9.67	5.76	7.82	4.91	6.85*	4.79*	7.13*	5.64*	<.001	0.12

^a^(*n* = 44) ^b^(*n* = 39).

*Significant difference relative to baseline.

*Abbreviations*: GAD-7, 7-item Generalized Anxiety Disorder Questionnaire; ISI, Insomnia Severity Index; PHQ-9, 9-item Patient Health Questionnaire.

#### Secondary outcome measures.

Mood. In acute insomnia participants, anxiety significantly improved in the short-term, as shown by the significant difference in GAD-7 scores between baseline and 1-week follow-up, with a large effect size (*t*(77) = 5.81; *p < *.001, *d*_*z*_ = 0.70; Table 2). Similarly, anxiety also significantly improved in the longer term, as shown by the significant main effect of time point (*F*(2.46, 105.73) = 7.05, *p < *.001; η^2^_p_ = 0.14; Table 3). *Post hoc* comparisons indicated that GAD-7 scores at all follow-up time points had significantly reduced relative to baseline.

Depression levels also significantly improved in the short-term, as shown by the significant difference in PHQ-9 scores between baseline and 1-week follow-up, with a large effect size (*t*(76) = 4.87; *p < *.001, *d*_*z*_ = 0.60; Table 2). Similarly, depression also improved in the longer term, as there was a significant main effect of time point (*F*(3, 114) = 5.24; *p < *.01; η^2^_p_ = 0.12; Table 3). *Post hoc* comparisons indicated that GAD-7 scores at one and 3-month follow-ups had significantly reduced relative to baseline.

Sleep continuity. There were no significant changes in subjective sleep continuity (TIB, TST, SE%, SOL, NWAK, or WASO) between the period before, and after, the intervention (all *p*-values > .008; Table 4).

**Table 4.** Acute Insomnia Subjective Sleep Continuity Comparisons: Pre-intervention and Post-Intervention (*n *= 50)

	Week 1		Week 2			
	Mean	*SD*	Mean	*SD*	*P*	*d*_*z*_
TIB (mins)^a^	708.06	243.30	673.73	215.78	.14	0.20
TST (mins)	434.93	68.78	444.30	60.28	.21	0.19
SOL (mins)	88.72	80.49	71.82	101.86	.25	0.24
NWAK	1.74	1.30	1.54	1.61	.18	0.18
WASO^a^ (mins)	63.73	100.44	43.92	67.52	.22	0.29
SE(%)^a^	66.35	16.11	70.49	14.96	.02	0.36

^a^(*n* = 49).

*Abbreviations*: NWAK, number of awakenings; SE, sleep efficiency; SOL, sleep onset latency; TIB, time in bed; TST, total sleep time; WASO, wake after sleep onset.

Good sleepers. In good sleepers, there was no significant change in insomnia severity in the short-term (at 1-week follow-up relative to baseline), as indicated by the lack of significant group (intervention or no intervention) × time point interaction (*F*(1, 63) = 2.76; *p > *.05; η^2^_p_ = 0.00; Table 5). Similarly, there was no significant change in insomnia severity in the longer term (at any follow-up time point relative to baseline), as indicated by the lack of significant group × time point interaction (*F*(2.31, 94.68) = .83; *p > *.05; η^2^_p_ = 0.02; Table 6). Additionally, there were no significant changes to anxiety or depression, either in the short-term, or longer-term, relative to baseline (both interaction *p*-values > .05; Table 5 and Table 6). Finally, when subjective sleep continuity was assessed in the good sleepers who had received the intervention, there were no other significant changes in subjective sleep continuity between baseline and follow-up (week 1 compared to week 2; all *p*-values > .008; Table 7).

**Table 5.** Good Sleeper Insomnia Severity and Mood Comparisons: Baseline and 1-Week Follow-up

	No intervention (*n *= 29)		Intervention (*n *= 27)			
	Mean	SD	Mean	SD	*P* (interaction)	η^2^_p_
ISI (baseline)	5.38	3.35	6.11	5.42	.65	0.00
ISI (follow-up)	5.03	3.99	5.14	4.90		
GAD-7 (baseline)^a^	5.70	4.20	6.00	4.97	.42	0.01
GAD-7 (follow-up)^a^	4.17	4.12	5.21	3.84		
PHQ-9 (baseline)^a^	5.10	4.15	6.21	5.13	.51	0.01
PHQ-9 (follow-up)^a^	4.37	3.76	6.04	4.63		

^a^(no intervention *n *= 30, intervention *n *= 24).

*Abbreviations*: GAD-7, 7-item Generalized Anxiety Disorder Questionnaire; ISI, Insomnia Severity Index; PHQ-9, 9-item Patient Health Questionnaire.

**Table 6.** Good Sleeper Baseline and Follow-up Comparisons: Insomnia Severity and Mood (*n *= 35)

	Baseline		1 week		1 month		3 months			
	Mean	*SD*	Mean	*SD*	Mean	*SD*	Mean	SD	*P*	η^2^_p_
ISI (no intervention)^a^	5.35	3.63	3.91	3.15	4.65	4.42	7.42	4.56		
ISI (intervention)^a^	6.75	6.09	5.42	4.06	7.42	4.56	7.75	5.22	.63	0.01
GAD-7 (no intervention)^b^	5.43	4.13	3.48	3.70	4.26	4.04	5.78	5.30		
GAD-7 (intervention)^b^	5.69	4.84	3.62	2.36	5.85	4.47	4.23	4.19	.22	0.04
PHQ-9 (no intervention) ^a^	4.78	4.34	3.04	2.98	4.09	4.91	5.96	4.63		
PHQ-9 (intervention) ^a^	6.25	6.33	5.58	5.53	6.75	5.10	7.08	6.14	.12	0.06

^a^(no intervention *n* = 23; intervention *n *= 12); ^b^(no intervention *n* = 23; intervention *n *= 13).

*Abbreviations*: GAD-7, 7-item Generalized Anxiety Disorder Questionnaire; ISI, Insomnia Severity Index; PHQ-9, 9-item Patient Health Questionnaire.

**Table 7.** Good Sleeper Subjective Sleep Continuity Comparisons: Pre/Post-intervention (*n *= 27)

	Week 1		Week 2			
	Mean	*SD*	Mean	*SD*	*P*	*d*_*z*_
TIB^a^ (mins)	648.71	166.29	670.73	164.50	.52	0.18
TST^b^ (mins)	488.69	50.03	502.16	50.84	.14	0.36
SOL^b^ (mins)	91.92	175.84	77.47	171.44	.32	0.11
NWAK^b^	0.90	0.58	0.71	0.79	.13	0.35
WASO (mins)^c^	38.85	76.93	18.91	36.46	.28	0.35
SE(%)^a^	75.22	14.01	77.74	14.40	.19	0.24

^a^(*n *= 25); ^b^(*n* = 26); ^c^(*n *= 24).

*Abbreviations*: NWAK, number of awakenings; SE, sleep efficiency; SOL, sleep onset latency; TIB, time in bed; TST, total sleep time; WASO, wake after sleep onset.

Remission and transition rates. A total of 146 (60.6%) acute insomnia participants displayed clinically significant ISI scores at baseline. A total of 17, 18, and 17 participants (26.2%, 34%, and 40.5% of acute insomnia participants at each time point) were classed as showing evidence of remission (classed as those who demonstrated a follow-up ISI score of < 10) at 1 week, 1 month and 3-month follow-up time points, respectively. Of the good sleeper group, a total of 3, 4, and 11 participants (3.8%, 6.6%, and 21.2% of those participants at each time point) transitioned to demonstrating clinically significant ISI scores (>10) at 1 week, 1 month and 3-month follow-up time points.

## Discussion

The main aim of the present study was to assess the effectiveness of an online behavioral self-help intervention for acute insomnia, in the context of the COVID-19 pandemic. The secondary aims of the study were to examine if the intervention could improve subjective sleep continuity and reduce anxiety and depression in acute insomnia, and, finally, to assess the feasibility and effectiveness of the intervention in good sleepers.

As was hypothesized, as well as the intervention being feasible, the results demonstrated that the intervention significantly reduced insomnia severity in acute insomnia participants in the short-term (1-week post-intervention), with a large effect size. Additionally, these beneficial effects were maintained at 1- and 3-month follow-up periods. In acute insomnia participants, similar results were observed for symptoms of depression, and anxiety, with large effect sizes. Despite this, the intervention did not appear to improve diary-derived sleep continuity measures. Overall, these results indicate that an online behavioral intervention can rapidly improve insomnia severity, and also improve mood, in individuals with acute insomnia. Importantly, these effects are maintained for up to 3 months following the intervention.

The results have implications for the treatment of acute insomnia. As demonstrated, this online behavioral intervention is rapidly effective for insomnia severity since positive effects were observed after only 1 week. As such, this intervention can be used as a non-pharmacological, low-cost, and easily accessible treatment for acute insomnia. Given the potential for this intervention to prevent the transition from acute insomnia to insomnia disorder [7], larger trials using this intervention are now warranted. Importantly, the use of an online approach may have specific advantages compared to face-to-face treatments. Although the self-help leaflet has previously been successfully used alongside face-to-face sleep restriction [40, 42, 45], the present study indicates that the self-help leaflet is effective in the treatment of acute insomnia in its own right. This is important as although brief CBT-I is highly effective in treating acute insomnia, online behavioral interventions offer specific advantages when compared to face-to-face CBT-I. Firstly, in-person CBT-I requires trained personnel, which has resource implications both in terms of staff time and cost. This is not the case with online interventions as practitioners can deliver internet-based CBT-I to more individuals than they can treat in-person, and internet-based interventions have good levels of acceptability [49, 66]. Secondly, online interventions have advantages in the context of situations such as the COVID-19 pandemic, as they can be used to treat individuals who have a fear of contamination or infection, and do not require the use of social distancing measures [67].

The present results are also important with regard to informing the design and delivery of future clinical trials for acute insomnia; particularly trials which intend to use online self-help interventions. Specifically, it was observed that for individuals with acute insomnia, the overall participant dropout rate was approximately 60% at 1 week, 72% at 1 month, and 79% at 3 months follow-up. Additionally, a significantly higher dropout rate was observed for acute insomnia participants who were allocated to the waitlist condition than those who were not, of approximately 71% at 1 week, 77% at 1 month, and 81% at 3-month follow-up time periods. Notably, the participant dropout levels observed in the present study are higher than the average attrition rate (approximately 50% of participants) shown in insomnia studies which have used full digital CBT-I [66]. However, meta-analytic evidence from trials of CBT in depression suggests that dropout rates of as high as 74% are observed when participants are provided with unsupported internet-based CBT [68]; the present study suggests that this is also the case in acute insomnia. Therefore, these results can be used to both inform a priori power analyses and for estimating participant retention at follow-up time points in future trials.

The present results can also inform the etiology of acute insomnia in the context of the COVID-19 pandemic. In the present study, not only did individuals with acute insomnia have significantly greater levels of subjective perceived stress at baseline than good sleepers, but they also showed greater levels of distress in relation to COVID-19 and a greater perceived level of vulnerability to infectious disease. Previous cross-sectional work has also suggested that the fear of infection may have a role in the development of sleep disturbances; one Chinese study has shown that sleep disturbances were more likely to occur in individuals who believed that COVID-19 had caused a high number of deaths, or that COVID-19 was not easy to cure, than individuals who did not believe this [26]. Additionally, other work has shown that concern in relation to catching COVID-19 can predict subjective stress levels [69] and that perceived stress is positively associated with COVID-19 severity [70]. Similarly, COVID-19-related worry (but not COVID-19 exposure) can predict the subsequent development of insomnia [71]. Together, these results suggest that distress relating to COVID-19, and the perceived vulnerability to COVID-19, could have a causal role in acute insomnia development, potentially due to increased perceived stress levels. This is in keeping with longitudinal work observing that sleep disturbances appear to occur alongside “waves” of COVID-19 cases [72, 73]. As the COVID-19 pandemic should be considered an ecologically valid and provocative stressor [30], this provides further support for the idea that acute insomnia should be considered stress-related [74].

The present study also demonstrates that the use of this intervention is feasible in good sleepers, and the observed participant dropout rates of 20%–50% can be used to inform the design of future studies with a sleep vaccination approach. Although in the present study, there was no effect upon subjective sleep continuity in good sleepers, it is likely that the intervention had less of an impact than the individual variations in sleep which have been caused by pandemic-related societal changes, where even in the absence of an intervention, sleep has actually *improved* in some individuals [73, 75]. This is potentially because of greater sleep opportunities in individual sleep schedules, caused by factors such as flexible working [76, 77]. Interestingly, it should be noted that there was a subjective trend towards a worsening in subjective insomnia severity, anxiety, and depression between the 1 and 3-month follow-up time points in good sleepers. Speculatively, this could suggest that some good sleepers used the intervention to *maintain* sleep in the short-term, but not in the longer-term. However, this possibility should be explored in more detail in future trials. Overall, as the present study shows that a sleep vaccination approach is now feasible, future studies should now design and test bespoke preventative sleep interventions for good sleepers.

The present study could be extended in several ways. Firstly, these positive results should be replicated in larger trials. Secondly, future work should determine how long the positive effects of the intervention last for. Although in the present study, the results indicated that the beneficial effects persisted at 3 months follow-up, “booster” interventions may be necessary to maintain these effects [42]. The examination of remission rates in those with acute insomnia who receive the intervention over longer periods of time is likely to be useful in determining the clinical significance of longer-term effectiveness. In the present study, 40% of poor sleeper participants showed remission during the 3-month period, and although this is lower than the rates observed in a prospective study, which observed remission rates of 72% at 1-year follow-up [11], this still demonstrates the clinical effectiveness of the intervention and this lower rate is likely to be due to the comparatively shorter follow-up period. Although it was beyond the scope of the present study to assess the proportion of acute insomnia participants who subsequently developed insomnia disorder, this would be of interest and should be investigated in adequately powered future trials. Thirdly, work should examine the mechanisms underpinning the beneficial effects of this intervention. As stress is likely to have a central role in causing acute insomnia [74], future work should investigate if the intervention can simultaneously reduce both subjective and objective stress. A relevant biological marker that could be used for this purpose is the stress hormone cortisol; cortisol is the end product of the hypothalamic-pituitary-adrenal (HPA) axis and is highly responsive to psychological stress, in a dose–response manner [78]. This measure is particularly suitable as HPA axis alterations are relevant to the pathogenesis of acute insomnia [30, 79], and as specific sleep research protocols exist for the measurement of cortisol, and particularly the cortisol awakening response [30, 80, 81], cortisol may be a useful biomarker of treatment effectiveness. Fourthly, work should examine if the effectiveness of this intervention can be improved through the provision of additional support, by monitoring or improving adherence.

The two main strengths of the study are that this study had a high level of ecological validity, by providing an indication of the effectiveness of online behavioral interventions for acute insomnia in a large-scale, representative, “real-world” situation [66]. Additionally, acute insomnia participants were defined as such on the basis of the DSM-5 diagnostic criteria [51], although we acknowledge that this was done on a self-report basis. However, despite the positive results and implications of the present study, there are four main limitations. Firstly, the participant dropout rate was higher than expected; however, this has important implications for future acute insomnia clinical trial design. Although the dropout rate could potentially be reduced by providing participants with additional support, as this can increase participant retention and improve treatment effectiveness [82, 83], this comes with additional costs. Future trials should assess if automated methods of trial support are feasible and can improve participant retention rates [49], or if there are specific participant characteristics which may predict or increase subsequent retention, as this was beyond the scope of the present study. Secondly, it was not possible to formally monitor treatment adherence in the present study, although this was partially mitigated by electronically verifying participant access to intervention leaflet. It is highly likely that there was a large variation in treatment adherence, where some participants only used the leaflet for a short period of time, and others used the leaflet on a more regular basis. However, this limitation is outweighed by the high levels of ecological validity. Thirdly, another potential limitation is that in the present study, an acute insomnia waitlist group was used as a comparison, instead of an active control group. Therefore, it is possible that potential placebo or nocebo effects might have influenced the results [84]. Although the use of a waitlist group provided important information regarding dropout rates, which can be used to inform the design of subsequent trials using this intervention, trials should also consider using established treatments (e.g. CBT-I) as a comparator. Similarly, future trials may wish to incorporate the use of intent-to-treat analyses, or use more sophisticated trial designs where all outcome measures are simultaneously assessed, to also minimize placebo or nocebo effects. Finally, the use of self-reported participant questionnaires as outcome measures have the potential to lead to response bias [85]; however, the ISI is a reliable and valid measure which is sensitive to treatment response [52].

Overall, this study indicates that an online behavioral self-help leaflet can rapidly reduce insomnia severity, and improve subjective anxiety and depression symptoms, in individuals with acute insomnia who engage with the intervention. Importantly, these beneficial effects are maintained at up to 3 months post-intervention. Larger trials using this intervention are now warranted.

## Supplementary Material

zsae059_suppl_Supplementary_Material

## Data Availability

The data underlying this article will be shared on reasonable request to the corresponding author.
